# Development of Certified Reference Material of L-Thyroxine by Using Mass Balance and Quantitative Nuclear Magnetic Resonance

**DOI:** 10.3390/molecules30132840

**Published:** 2025-07-02

**Authors:** Qiang Zhao, Weifei Zhang, Dan Song, Xirui Zhou, Xianjiang Li, Huan Yao, Wenjing Xing, Hongmei Li, Jian Ma, Peng Xiao

**Affiliations:** 1 Department of Immunology, Second Affiliated Hospital of Harbin Medical University, Harbin 150081, China; zq3231@126.com (Q.Z.); xingwenjing1984@126.com (W.X.); 2 Division of Chemical Metrology and Analytical Science, National Institute of Metrology, Beijing 100029, China; zhangweifei@nim.ac.cn (W.Z.); songdan@nim.ac.cn (D.S.); zhouxr@nim.ac.cn (X.Z.); lixianjiang@nim.ac.cn (X.L.); yaohuan@nim.ac.cn (H.Y.); lihm@nim.ac.cn (H.L.); 3 Key Laboratory of Chemical Metrology and Applications on Nutrition and Health, State Administration for Market Regulation, Beijing 100029, China

**Keywords:** L-thyroxine, certified reference material, mass balance, quantitative nuclear magnetic resonance spectroscopy, traceability

## Abstract

L-thyroxine (T4) is an important hormone for diagnosing and evaluating thyroid function disorders. As outlined in ISO17511, having a certified reference material (CRM) is crucial for ensuring that the results of clinical tests are traceable to the SI-unit. This study employed two principal methods to evaluate the purity of T4, mass balance (MB) and quantitative nuclear magnetic resonance (qNMR), both of which are SI-traceable (International System of Units) approaches. The MB method involved a detailed analysis of impurities, including water, structurally related compounds, and volatile and non-volatile substances. A variety of techniques were employed to characterize T4 and its impurities, including liquid-phase tandem high-resolution mass spectrometry, ultraviolet spectrophotometry, infrared spectroscopy, and both ^1^H-NMR and ^13^C-NMR. Additionally, impurities were quantified using Karl Fischer coulometric titration, ion chromatography, gas chromatography–mass spectrometry, and inductively coupled plasma–mass spectrometry. In qNMR, ethylparaben was used as the internal standard for direct value assignment. The results showed T4 purities of 94.92% and 94.88% for the MB and qNMR methods, respectively. The water content was determined to be 3.563% (n = 6), representing the highest impurity content. Ten structurally related organic impurities were successfully separated, and five of them were quantified. Ultimately, a purity of 94.90% was assigned to T4 CRM, with an expanded uncertainty of 0.34% (*k* = 2).

## 1. Introduction

L-thyroxine (T4, CAS: 51-48-9) plays an important role in regulating development, growth, and energy metabolism, and its insufficient or excessive secretion can cause diseases [1,2,3]. Therefore, the serum or plasma T4 concentration can be used as an indicator for the diagnosis of thyroid diseases. In the light of the compelling evidence provided by Li et al., at least 200 million people were diagnosed with thyroid nodules and other diseases in Mainland China [4]. These numbers highlight the need for increased awareness and further action to address thyroid disease. This situation motivates us to develop T4 CRM to fulfill the increasing demand for thyroid function diseases.

The standard ISO17511:2020 has been widely adopted in developing in vitro diagnostic (IVD) reagents [5]. This standard outlines the technical requirements to establish metrological traceability for values assigned to calibrators, trueness control materials, and human samples used with IVD medical devices. There are six traceability models involved in ISO17511:2020. When primary certified reference materials (CRMs) and reference measurement procedures (RMPs) are available, the standard value can be assigned to calibrators to establish the metrological traceability [6]. In this context, T4-purity CRM is the indispensable benchmark for ensuring accurate and comparable clinical measurements. The Institute for Reference Materials and Measurements (IRMM) pioneered the development of an SI-traceable T4 CRM globally, designated as IRMM 468. Moreover, the Laboratory of the Government Chemist (LGC) and Dr. Ehrenstorfer have also developed a high-purity, SI-traceable CRM, designated as DRE-C17575300. The certified value of IRMM 468 is the purity after considering inorganic residues, water, ethanol, and organic impurities detectable by HPLC-UV and HPLC-MS. The certified value is traceable to the International System of Units (SI). The introduction of IRMM 468 has substantially advanced the standardization of research in thyroid function tests [7,8].

However, in recent years, IRMM 468 has been frequently unavailable, thereby failing to meet the traceability requirements for IVD in China. Mass balance (MB) [9] and quantitative nuclear magnetic resonance (qNMR) [10] are widely used primary measurement approaches for the purity assessment of organic materials. MB is an indirect method that can be traced back to the SI unit. By this strategy, the mass fraction can be calculated by deducting all of the impurities from 100% or 1000 mg/g, and IRMM 468 was quantified in this way. While qNMR is a direct method, the mass fraction is assigned with a well-characterized SI-traceable standard. It should be noted that, prior to the selection of quantitative hydrogen atoms, an investigation into the major structurally related impurities using NMR is required, as the overlap in hydrogen chemical shifts from the target compound and impurities may reduce quantitative accuracy.

This work implemented an extensive purity assessment of T4 with a combination of MB and qNMR approaches for the first time. In the MB approach, structurally related impurities were determined by high-performance liquid chromatography–tandem ultraviolet detector (HPLC-UV) and partly identified by HPLC–tandem high-resolution mass spectrometry (HPLC-hrMS). The other impurities (water, volatile, and residual inorganic substances) were quantified by Karl Fischer coulometric titration (KFC), gas chromatography–mass spectrometry (GC-MS), ion chromatography (IC), and inductively coupled plasma–mass spectrometry (ICP-MS), respectively. In the qNMR approach, the purity was calculated by the selected hydrogen peaks from T4 and the internal standard (ethylparaben). This T4 CRM was traceable to the SI, and the associated uncertainty contribution of the two methods was carefully evaluated. Compared to previous research on IRMM 468, this study has broadened the quantitative analysis of structurally related impurities and the application of the qNMR method for determining the purity of T4. The purpose is to enhance the reliability of the CRM.

## 2. Results and Discussion

### 2.1. Qualitative Characterization

#### 2.1.1. The Measurand

The purpose of qualitative characterization was to verify the identity of T4 material. In Figure S1, the characteristic absorption peaks in the IR spectrum can be explained by typical functional groups. In detail, 3509 cm^−1^ was attributed to O-H stretching vibration, 1608 cm^−1^ was attributed to C=O stretching vibration, 1583 cm^−1^ and 1183 cm^−1^ to N-H asymmetric and symmetric stretching vibration, and 1238 cm^−1^ to Ether-O-stretching vibration. In Figure S2, the UV spectrum showed that the significant absorptions were located at 195 nm and 225 nm. Such results were in accordance with a previous report [11]. However, since the UV wavelength is closely related to the value assignment in MB method, the wavelength needs to be carefully characterized ahead quantitative research.

**Figure 1:**
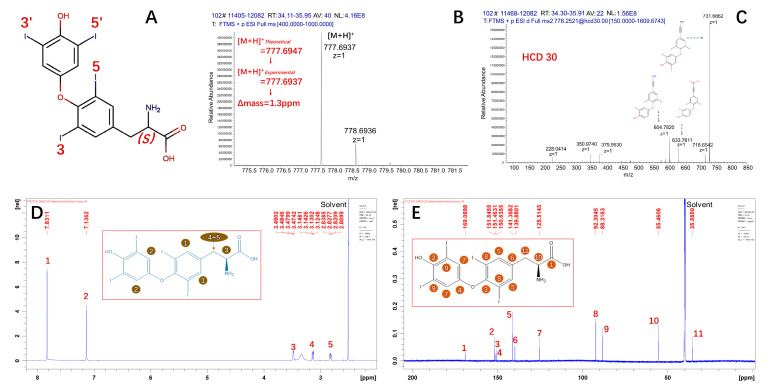
Characterization of T4: (**A**) chemical structure, (**B**) mass spectrum of T4 CRM, (**C**) MS/MS spectrum of T4, (**D**) ^1^H-NMR spectrum of T4 CRM in DMSO-*d*_6_, and (**E**) ^13^C-NMR spectrum of T4 CRM in DMSO-*d*_6_.

Figure 1A shows the chemical structure of T4. The mass spectrum in Figure 1B indicates that the monoisotopic precursor ion of T4 was *m*/*z* 777.6937. The mass deviation was 1.3 ppm based on the theoretical value. Afterward, HCD fragmentation was utilized to generate productions (Figure 1C). The production of *m*/*z* 731.6862, which corresponds to the ion resulting from the loss of formic acid, was the base peak ion when 30 V of energy was applied. Meanwhile, the ion of *m*/*z* 604.7820 corresponds to losses of formic acid and iodine radical.

As shown in Figure 1D, the hydrogen chemical shifts were H-1 (7.83 ppm, 2H), H-2 (7.14 ppm, 2H), H-3 (3.48 ppm, 1H), and H-4 and 5 (3.14 ppm, 1H; 2.83 ppm, 1H). Additionally, the chemical shift of residual solvent in dimethyl sulfoxide-*d*_6_ was 2.51 ppm. As shown in Figure 1E, the carbon chemical shifts were C-1 (169.1 ppm, 1C), C-2 (151.8 ppm, 1C), C-3 (151.4 ppm, 1C), C-4 (150.6 ppm, 1C), C-5 (141.3 ppm, 2C), C-6 (139.8 ppm, 1C), C-7 (125.5 ppm, 2C), C-8 (92.3 ppm, 2C), C-9 (88.3 ppm, 2C), C-10 (55.4 ppm, 1C), and C-11 (35.5 ppm, 1C). The chemical shifts in ^13^C NMR spectrum of T4 CRM were in accordance with a previous report [12].

These qualitative results indicated that the main component of candidate material was T4.

#### 2.1.2. Structural-Related Impurities

During the synthesis, purification, storage, and transportation of the T4 CRM, some structural-related organic impurities might be generated. Therefore, identification of them as much as possible is the prerequisite for an accurate value assignment in MB quantification. The conditions in liquid chromatographic separation, including ultraviolet wavelength, liquid-phase composition, column temperature, and elution time, would have an evident impact on impurity analysis. Concerning parameters like UV wavelength [11], analytical column, stationary phase [13], and mobile phase [14], the reader could be referred to the previous reports. The further characterizations of structural-related impurities are presented in the Supplementary Materials. It is evident that no additional impurities were detected with an extended separation time (Figure S3) or an elevated column temperature (Figure S4).

**Figure 2:**
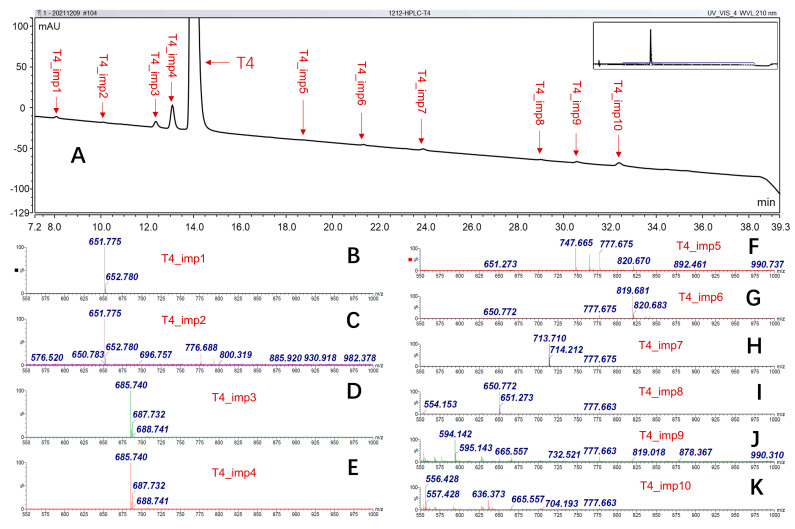
Characterization of the structurally related impurities involved in T4 CRM. (**A**) The LC chromatography. (**B**–**K**) Mass spectrum of each impurity.

As shown in Figure 2A, a reliable separation of structural-related impurities was achieved by using the optimized LC conditions. Each mass spectrum in Figure 2B–K corresponded to a substance. It can be seen that two pairs of isomers are involved in T4 CRM (Figure 2B–E). Since the structurally related impurities were mainly generated in the synthesis process, understanding the production process is helpful for us to identify them. There are two distinct multi-step routes to produce T4 to date. One way is to start with L-tyrosine nitration, followed by N-acetylation and then esterification to give 3,5-dinitro-N-acetyl-L-tyrosine ethyl ester. The resulting compound underwent coupling with p-methoxyphenol to give the corresponding diphenyl ether, which upon hydrogenation, results in diamine. Diamine compound, upon iodination via diazotization, demethylation, hydrolysis, and finally ring iodination, gives L-thyroxine [15]. Another way is to start with an iodination reaction of L-tyrosine to generate 3,5-diiodo-L-tyrosine. Subsequently, the amino and carboxyl groups of 3,5-diiodo-L-tyrosine were protected, and a key intermediate (N-acetyl-3,5-diiodo-L-tyrosine ethyl ester) was formed. After self-coupling of the intermediate, the coupling product was hydrolyzed using an acid solution, which involved a mixture of hydrochloric acid and acetic acid. The latter one is also called biomimetic synthesis via intramolecular coupling [16]. L-thyroxine synthesis development is well reviewed by the Reddy group [17].

**Figure 3:**
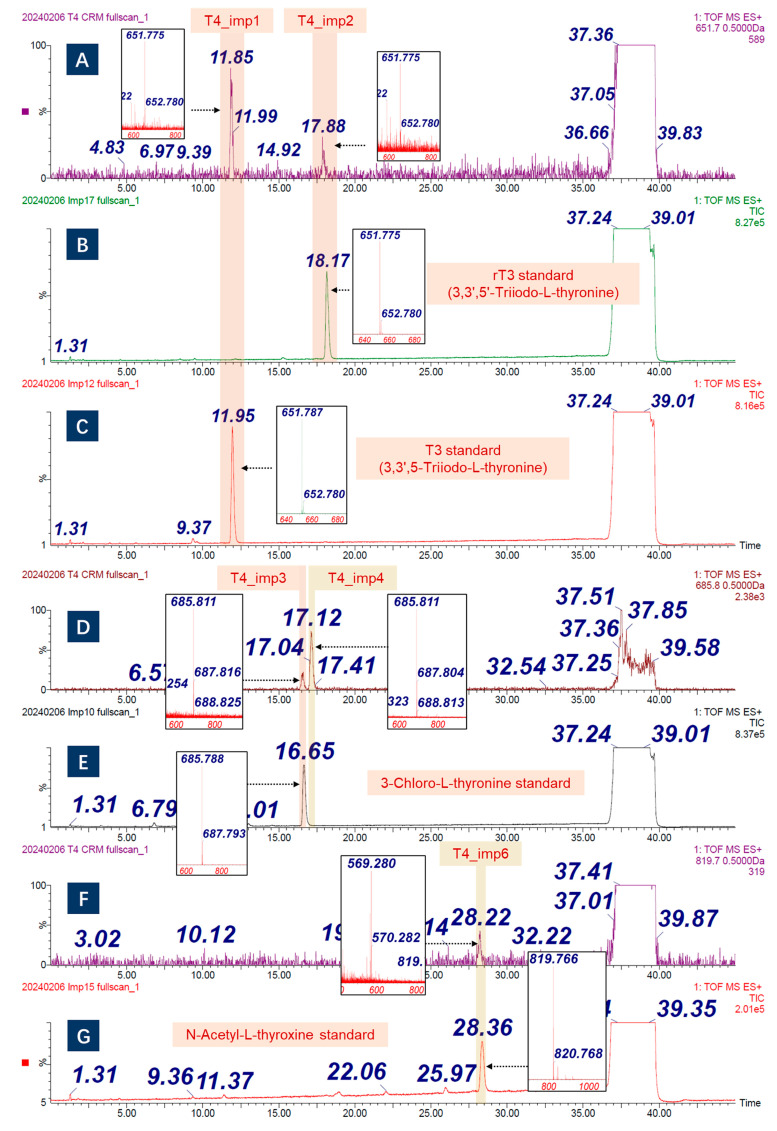
Identification of T4 structurally related impurities by MS method. (**A**) Extracted Ion Chromatogram (EIC) of *m*/*z* 651.775 from a Total Ion Chromatogram (TIC) of T4 CRM, corresponding to T4_imp1 and T4_imp2. (**B**,**C**) The retention times and *m*/*z* values of 3,3′,5′-Triiodo-L-thyronine and 3,3′,5-Triiodo-L-thyronine standards. (**D**) EIC of *m*/*z* 685.811 from a TIC of T4 CRM, corresponding to T4_imp3 and T4_imp4. (**E**) the retention time and *m*/*z* value of 3-chloro-L-thyronine standards. (**F**) EIC of *m*/*z* 819.766 from a TIC of T4 CRM, corresponding to T4_imp6. (**G**) the retention time and *m*/*z* value of N-acetyl-L-thyroxine standard.

As the molecular weight differs by −125.9 Da, T4_imp1 and T4_imp2 might be the deiodination product of T4. Furthermore, two highly pure Triiodo-L-thyronine chemicals, 3,3′,5-Triiodo-L-thyronine (T3) and 3,3′,5′-Triiodo-L-thyronine (rT3), were analyzed with identical LC conditions. The measured *m*/*z* and retention times in Figure 3A–C indicate that T4_imp1 and T4_imp2 are T3 and rT3, respectively. T4_imp3 and T4_imp4 performed an exceptional pattern of isotope peak distribution (Figure S6), indicating that chlorine atoms might be involved [18]. Since the intermediate needs to be hydrolyzed by hydrochloric acid, the iodine has a chance to be replaced by chlorine. Then, a highly pure chloro-l-thyroxine (3-chlolo-L-thyronine) was adopted to confirm the supposition. Based on the *m*/*z* and LC retention times (Figure 3D,E), the T4_imp3 was identified as 3-chlolo-L-thyronine. Meanwhile, T4_imp3 was also known as Levothyroxine EP Impurity B. However, the substitution site of T4_imp4 was unclear. Here, it was defined as X-chloro-L-thyronine. According to the product ion distribution of T4_imp5, it was likely to be the acetamide impurity. As shown in Figure S7, the precursor ion (*m*/*z* 747.701) was fragmented then to form product ions like *m*/*z* 731.707 (ions of loss of oxygen or NH_2_ radical), *m*/*z* 620.796 (deiodination ions), and *m*/*z* 576.776. As mentioned above, the acetylation reaction was implemented on the amino group of intermediates before intramolecular coupling. Therefore, if the acetyl group was not fully hydrolyzed, acetylation-modified impurity might be present. The MS data showed a mass difference of +42 Da between T4_imp6 and T4. Furthermore, when employing a high-pure N-acetyl-L-thyroxine as standard, the retention times were identical (Figure 3F,G). T4_imp7 and T4_imp8 were the higher-molecule-weight compounds, which might be the dimer pattern. Based on the previous report [13], the two impurities were the biphenyl-bridged thyronine. The T4_imp9 and T4_imp10 were the unknown molecules, and no published documents have discussed them. The results of structurally related impurities are summarized in Table 1.

#### 2.1.3. Enantiomer in CRM Candidate

Some studies have suggested that derivatized cyclofructan may serve as an effective stationary phase for the separation of thyroxine enantiomers, and a mixture of hexane and ethanol can be utilized as the mobile phase [19,20,21]. As shown in Figure S8, a relatively satisfactory separation of L-T4 and D-T4 was achieved when 30% ethanol in hexane was employed as the mobile phase, although baseline resolution was not fully attained. Apparently, the spectrum presented in Figure S9 cannot conclusively serve as evidence to verify whether any D-T4 is present in the L-T4 CRM.

Furthermore, the specific optical rotation of T4 CRM, which constitutes another critical method for investigating potential D-T4 impurities, was assessed using a polarimeter. As reported in a prior study, the specific rotation of L-T4 at a concentration of 3.3% was −4.4° (20 °C, with a wavelength of 546 nm and a light path length of 100 mm) when dissolved in a solvent consisting of 70% methanol solution containing 0.13 N NaOH [22]. Accordingly, the samples prepared for the determination of specific optical rotation were rigorously aligned with these experimental conditions and parameters. As presented in Table S1, the values obtained for sample 1 were consistent with those documented in the literature [22]. Thyroxine in sample 2 contained one ten-thousandth of D-T4 but exhibited no significant deviation in its optical rotation values. In contrast, thyroxine in sample 3 contained one thousandth of D-T4, significantly higher than that in sample 2; however, no substantial variation was observed in the specific optical rotation values.

Although there is currently no definitive evidence to confirm that all molecules in T4 CRM are levothyroxine, based on the LC characterization results and the determined values of specific optical rotation, it can be concluded that the relative content of D-T4 does not exceed 0.01% (*w*/*w*). Therefore, the compound in T4 CRM may be regarded as levothyroxine.

**Table 1:** Summary of the structurally related impurities involved in T4 CRM.

Code	Mono. M.W. (Da)	Compound	CAS	High-Pure Material	Validated by			Content
					LC	MS	Ref.	
T4_imp1	650.7	3,3′,5-Triiodo-L-thyronine	6893-02-3	√	√	√	[23]	0.057%
T4_imp2	650.7	3,3′,5′-Triiodo-L-thyronine	5817-39-0	√	√	√	[23]	0.019%
T4_imp3	684.8	3-chloro-L-thyronine	1628720-66-0	√	√	√	/	0.187%
T4_imp4	684.8	X-chloro-L-thyronine	/	√	/	√	/	0.702%
T4_imp5	746.6	Acetamide	176258-88-1	/	/	√	/	/
T4_imp6	818.7	N-acetyl-L-thyroxine	26041-51-0	√	√	√	/	0.041%
T4_imp7	1425.4	T3−T4 dimer (biphenyl-bridged)	/	/	/	√	[13]	/
T4_imp8	1299.5	T3−T3 dimer (biphenyl-bridged)	/	/	/	√	[13]	/
T4_imp9	593.1	/	/	/	/	√	/	/
T4_imp10	555.4	/	/	/	/	√	/	/

### 2.2. Homogeneity and Stability

The results of the homogeneity test conducted on T4 CRM are presented in Table S2. According to the *F*-test for analysis of variance, the calculated *F* value of 2.03 was smaller than the critical *F* value of 2.31, indicating that there were no statistically significant differences between bottles and within bottles. Therefore, the CRM units prepared in this work were sufficiently homogeneous. 

Stability can indicate the tendency of characteristic values of the CRM to change under storage and transportation conditions. The test results, including standard deviation, s($\beta_{1}$), were calculated using *t*-test according to the ISO 33405 [24]. The slopes of the lines are depicted in Figures S10 and S11. The absolute values of the slopes were regarded as statistically insignificant when |$\beta_{1}$ | < *t*_0.95, n−2_⋅s($\beta_{1}$), with a confidence level of 95% and a coverage factor of 2. As shown in Tables S2 and S3, the values of |$\beta_{1}$| were smaller than the product of *t*_0.95, n–2_ and s($\beta_{1}$), confirming the excellent stability of T4 CRM.

### 2.3. Quantitative Analysis by MB

#### 2.3.1. Structurally Related Impurity Determination

The trace amounts of impurity in pure materials may go undetected because of inherent limitations of the detector used. Here, it can be observed that the baseline noise was about 0.01 mAu (Figure S12), and the limit of quantification of the UV detector was ten times the noise (0.1 mAu). Based on the prior characterization of the LC method, a signal intensity of 0.1 mAu corresponds to an injection amount of approximately 0.15 ng of thyroxine. For instance, when CRM concentration was prepared at 1250 ng/μL and 2 μL (2500 ng) of it was injected, the impurities with a content of more than 0.006% could be detected and quantified theoretically.

According to the preliminary results, the impurity with the lowest relative content (T4_imp5) was about 2 ng when 2500 ng of CRM was analyzed, which could be greater than the quantitation limits. Another challenge was achieving baseline separation between the main components and the impurities [25,26]. The spectrum in Figure S13 indicates that the main component partially overlapped with T4_imp4 when the injection amount was more than 1000 ng. Similarly, such a situation also occurred on T4_imp6 and T4_imp7, and T4_imp8 and T4_imp9. Therefore, 1000 ng of CRM was adopted in impurities quantification.

As summarized in Table 1, T4_imp1, T4_imp2, T4_imp3, T4_imp4, and T4_imp6 can be quantified by an external standard method because they were identified and the corresponding standards or their structural isomers were available. The UV adsorptions of each impurity were scanned by an SPD-20A UV/Vis LC detector (Figure S14). The wavelengths were utilized to plot the standard curves (Figure S15), and the content could be calculated based on each regression equation (Table 1). Furthermore, Equation (1) was employed in the MB method because not all the structurally related impurities can be quantified using standards. Therefore, the unidentified impurities (T4_imp9 and T4_imp10) and impurities without standards (T4_imp5, T4_imp7, and T4_imp8), could be subtracted by chromatographic peak area normalization method (Table 2). The organic purity (*P_o_*) calculation approach—some researchers abbreviated it to the LC-UV, LC-PDA, or HPLC approach—was widely adopted in purity reference material development [27,28].

#### 2.3.2. Water Determination

The blank value was determined with a dummy addition to compensate for the adventitious water introduced during sample loading, which was then subtracted from the mass of water determined in the T4 CRM sample. The data in Figure S16A suggests that the instrument is reliable. The six independent measurements determined the water content to be 3.563%, with an RSD of 2.018%. The water content was estimated in rectangular distribution, and the standard uncertainty was 0.125%. Since the water content is relatively high, the stability of the water content was further investigated. Furthermore, an additional characterization was carried out to monitor the trend of water content variation. As shown in Figure S16B, although there were slight changes in its content after unsealing (environmental conditions: relative humidity, 28.7%; temperature, 22.8 °C), all the results did not exceed the range of uncertainty. Such investigation could guide users to use CRM reasonably.

#### 2.3.3. Inorganic Impurity Determination

A semi-quantitative analysis mode was performed for preliminary screening for the inorganic elements. As shown in Table S4, the total content of inorganic impurity was only 0.006% by the ICP-MS approach. According to the chemical synthesis pathway, the residual counter ions might be involved. Verification measurements by ion chromatography confirmed the presence of counter ions for T4 CRM. The overlapping spectra of anion standards and T4 CRM samples are shown in Figure S17. The counter ions were detected, including acetate, formate, chloride, nitrate, and sulfate. Based on the external standard method, the total content of counter ions was 0.132% (Table S5).

#### 2.3.4. Residual Organic Solvent Determination

The residual organic solvent was determined by the headspace GC-MS method. The solvent of the T4 material was tested as a blank sample (Figure S18A). Figure S18B is the chromatogram of mixed standard organic solvent. After background subtraction, the results showed that no detectable organic solvent was present in the T4 CRM (Figure S18C).

#### 2.3.5. Mass Fraction by MB

According to Equation (1) [29], the purity of T4 CRM was calculated by deducting the content of all impurities from 100%, and Po was obtained by the LC-PDA approach. At last, the purity was determined to be 94.92%.

(1) $$P_{T4\_MB}=P_{O}(100\%-X_{RS}-X_{W}-X_{V}-X_{NV})$$

where $P_{T4\_MB}$, $P_{O}$, $X_{RS}$, $X_{W}$, $X_{V}$, and $X_{NV}$ are the purity of T4 CRM determined by MB (%), the content of T4 determined by LC-UV (%), the content of T4 structural-related impurities (%), the content of the moisture (%), the contents of volatile impurities (%), and the contents of residual inorganic impurities (%), respectively.

### 2.4. Quantitative Analysis by ^1^H qNMR

**Figure 4:**
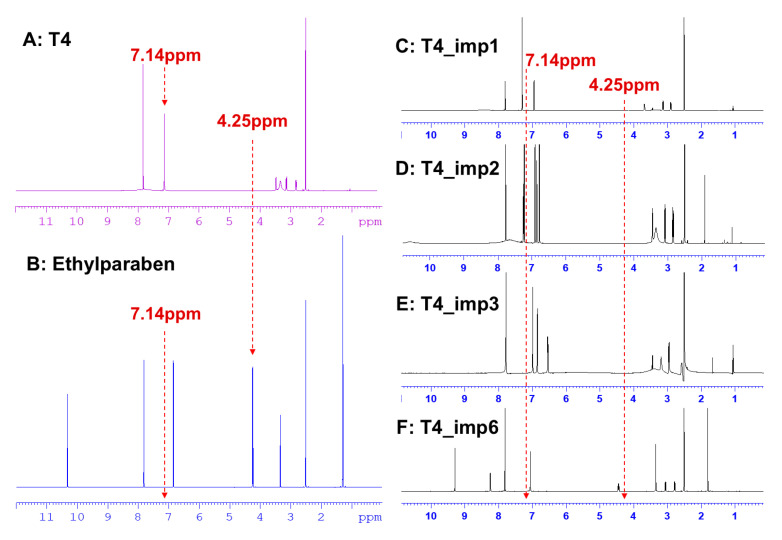
^1^H-NMR characterization of (**A**) T4 CRM, (**B**) internal standard, (**C**) T4_imp1 standard, (**D**) T4_imp2 standard, (**E**) T4_imp3 standard, and (**F**) T4_imp6 standard.

qNMR provided an independent, absolute quantitative method for CRM purity assessment. It overcame the problem of impurities not being detected by MB. For the qNMR method, an SI-traceable internal standard CRM was necessary for accurate quantification. As shown in Figure 4A,B, the selected hydrogen chemical shifts of ethylparaben and T4 had no overlaps (7.14 ppm vs. 4.25 ppm). Meanwhile, the hydrogen chemical shifts of impurities were also taken into account as a factor in the selection of internal standards. In Figure 4C–F, the available impurity standards were analyzed by ^1^H-NMR. The chemical shifts of the four impurities had no overlap with ethylparaben at 4.25 ppm. Therefore, the SI-traceable CRM of ethylparaben was selected as the internal standard. 

The purity of T4 was directly assigned to ethylparaben CRM by relative integral ratios of the selected hydrogen signals to them based on Equation (2). The result was 94.88%, with an RSD of 0.049% in six duplicates.

(2) $$P_{T4\_qNMR}=\frac{I_{T4}}{I_{std}}\cdot \frac{N_{std}}{N_{T4}}\cdot \frac{M_{T4}}{M_{std}}\cdot \frac{m_{std}}{m_{T4}}\cdot P_{std}$$

where $P_{T4\_qNMR}$ is the purity (%) of the T4 CRM; $I_{T4}$ is the integrated signal response of the measurand; $I_{std}$ is the integrated signal response of the internal standard; $N_{T4}$ is the spin number of the measurand; $N_{std}$ is the spin number of the internal standard; $M_{T4}$ is the molar mass (g/mol) of the T4; $M_{std}$ is the molar mass (g/mol) of the internal standard; $m_{T4}$ is the weight (g) of the T4; $m_{std}$ is the weight (g) of the internal standard; and $P_{std}$ is the purity (%) of the internal standard.

### 2.5. Uncertainty Evaluation

#### 2.5.1. Uncertainty of Homogeneity

The values of $s_{1}^{2}$ and $s_{2}^{2}$ are listed in Table S2. The value of the variable n represents the number of measurements. Since ANOVA suggested that the between-bottle variance is higher than the within-bottle variance, a calculation was performed in accordance with Equation (3) to determine the uncertainty of homogeneity.

(3) $$u_{bb}=s_{bb}=\sqrt{\frac{s_{1}^{2}-s_{2}^{2}}{n}}$$

The uncertainty related to the homogeneity of the data is represented by the symbol $u_{bb}$.

#### 2.5.2. Uncertainty of Stability

The uncertainty of stability ($u_{stability}$) was calculated based on Equation (4), which includes the uncertainties of both short-term stability, $u_{sts}$, and long-term stability, $u_{lts}$.

(4) $$u_{stability}=s\left(\beta_{1}\right)\times x$$

The uncertainty associated with the slope, designated as $s\left(\beta_{1}\right)$ (Tables S2 and S3), is a function of the monitoring period. x represents the monitoring time (7 days or 36 months). The uncertainties of the short-term stability were 0.0092% (4 °C, 7 days) and 0.0094% (20 °C, 7 days), which are almost identical. The uncertainty of the short-term stability was 0.005%. The uncertainties are listed in Table 3.

#### 2.5.3. Uncertainty of the MB Method

The combined standard uncertainty ($u\left(P_{MB}\right)$) was obtained by quadratic combination of the uncertainties from all detectable impurity, as shown in Equation (5) [30]:

(5) $$u\left(P_{MB}\right)=P_{MB}\cdot \sqrt{{\left[u_{\begin{array}{c} rel \end{array}}\left(P_{o}\right)\right]}^{2}+\frac{{\left[u\left(X_{RS}\right)\right]}^{2}+{\left[u\left(X_{W}\right)\right]}^{2}+{\left[u\left(X_{V}\right)\right]}^{2}+{\left[u\left(X_{NV}\right)\right]}^{2}}{{\left(1-X_{RS}-X_{W}-X_{V}-X_{NV}\right)}^{2}}}$$

where $u_{\begin{array}{c} rel \end{array}}\left(P_{o}\right)$ is the relative uncertainty from LC-UV (0.0011%), $u\left(X_{RS}\right)$ is the uncertainty from quantified structurally related impurities (0.0085%), $u\left(X_{W}\right)$ is the uncertainty from water (0.125%), $u\left(X_{V}\right)$ is the uncertainty from residual organic solvent (0%), and $u\left(X_{NV}\right)$ is the uncertainty from inorganic impurity (0.132%). In this way, the uncertainty of MB was assessed with 0.186%. Since the results of elemental impurities measured by ICP-MS were negligible (Figure S4), the uncertainty associated with inorganic impurities was primarily attributed to the quantification of counter ions. Given the substantial impact of environmental factors and instrumentation on the determination of trace impurities, the uncertainty introduced in this context will be conservatively estimated as 100% of the measured content (Figure S5).

#### 2.5.4. Uncertainty of the qNMR Method

For qNMR, the uncertainty budget of CRM was 0.281%, originating from Equation (6) [30] below:

(6) $$u_{rel}\left(P_{qNMR}\right)=\sqrt{{\left[\frac{u\left(\frac{I_{x}}{I_{std}}\right)}{\frac{I_{x}}{I_{std}}}\right]}^{2}+{\left[\frac{u\left(M_{x}\right)}{M_{x}}\right]}^{2}+{\left[\frac{u\left(M_{std}\right)}{M_{std}}\right]}^{2}+{\left[\frac{u\left(m_{x}\right)}{m_{x}}\right]}^{2}+{\left[\frac{u\left(m_{std}\right)}{m_{std}}\right]}^{2}+{\left[\frac{u\left(P_{std}\right)}{P_{std}}\right]}^{2}}$$

The two methods were determined to have equal precision since the calculated *F* value was below the threshold (Table 2). Meanwhile, the result of the *t*-test analysis, calculated using Equation (7), was 1.6, which is lower than the threshold value of *t*_0.95,12_ (2.18). Such a situation indicates that the purity values assigned by both methods were consistent.

(7) $$t=\frac{\left|\bar{x_{1}}-\bar{x_{2}}\right|}{\sqrt{\frac{\left(n_{1}-1\right)s_{1}^{2}+\left(n_{2}-1\right)s_{2}^{2}}{n_{1}+n_{2}-2}\times \frac{\left(n_{1}+n_{2}\right)}{n_{1}n_{2}}}}$$

Therefore, the standard uncertainty ($u_{char}$) of characterization was half of the square root of the sum of squares of purity uncertainty from value assignment of two methods, as shown in Equation (8):

(8) $$u_{char}=\sqrt{{\left(\frac{u_{MB}}{2}\right)}^{2}+{\left(\frac{u_{qNMR}}{2}\right)}^{2}}$$

Finally, the relative standard uncertainty of T4 CRM characterization was 0.168%. Details are described in Table 3. The expanded uncertainty, *U*, was calculated by multiplying the $u_{CRM}$ with coverage factor (*k* = 2) at the confidence level of 95%. Finally, the relative expanded uncertainty of the T4 CRM characterization was 0.336% (*k* = 2).

## 3. Materials and Methods

### 3.1. Chemicals and Materials

T4 candidate material was purchased from Aladdin (Shanghai, China). LC-MS-grade water, methanol (MeOH), acetonitrile (ACN), and formic acid (FA) were purchased from Fisher Chemicals (Ottawa, ON, Canada). Stock solution of T4 was prepared at the concentration of 1 mg/g with H_2_O (0.5% NH_4_OH, *v*/*v*) and stored at −20 °C in the dark. KBr for infrared (IR) and D-thyroxine (D-T4) (CAS:51-49-0) for isomer analysis were supplied by Aladdin (Shanghai, China). Dimethyl sulfoxide-*d*_6_ (DMSO-*d*_6_) was purchased from Cambridge Isotope Laboratories (Andover, MA, USA). Water content (GBW13515, 156.3 ± 1.3 mg/g) and ethylparaben (GBW06120, 99.97% ± 0.05% (*k* = 2)) CRMs were from National Institute of Metrology (Beijing, China). 3,3′,5-Triiodo-L-thyronine (CAS: 6893-02-3) was purchased from Sigma-Aldrich (Saint Louis, MO, USA), 3,3′,5′-Triiodo-L-thyronine (CAS: 5817-39-0) was purchased from TargetMol (Boston, MA, USA), 3-chloro-L-thyronine (CAS: 1628720-66-0) was purchased from ZZ Standard (Shanghai, China), and N-acetyl-L-thyroxine (CAS: 26041-51-0) was purchased from Cayman Chemical (Ann Arbor, MI, USA).

### 3.2. Instruments

HPLC-UV was performed on a ThermoFisher Vanquish equipped with Waters ACQUITY BEH C18 column (2.5 μm, 2.1 × 100 mm). The U3900 UV spectrophotometer (HITACHI, Tokyo, Japan) and SPD-20A UV/V, a liquid chromatography detector (Shimadzu, Tokyo, Japan), were used for main component and structurally related impurities adsorption characterization. HPLC-hrMS was performed on Waters UPLC Synapt G2 system (Manchester, UK) and ThermoFisher UHPLC Orbitrap Exploris^TM^ 240 system (Bremen, Germany). Rudolph (Autopol V Plus AutoFill, Hackettstown, NJ, USA) polarimeter was employed for enantiomer characterization. T4 stock solution was injected into a DB-624 (60 m × 0.250 mm × 1.40 μm) column (Agilent J&W, CA, USA) on Shimadzu GCMS-QP2020NX with HS-20NX headspace injector (Tokyo, Japan). Karl Fischer coulometric titrator was from Mettler-Toledo C30S (Greifensee, Switzerland). ICP-MS was on Agilent 8800 (Tokyo, Japan). IR was tested on PerkingElmer 100 N (Waltham, MA, USA). Ion chromatography (IC) was operated on Dionex ICS-5000+ HPIC system (ThermoFisher, Waltham, MA, USA). H-NMR and C-NMR were collected on Bruker AscendTM 800 NMR spectrometer (Billerica, MA, USA) installed with quadruple inverse 5 mm CPQCI cryo-probe. Sample was weighed by Mettler-Toledo XP205 or UMX2 balance (Greifensee, Switzerland).

### 3.3. Qualitative Characterization

#### 3.3.1. IR Analysis

The powder of KBr was dried in advance and milled with T4 to form a homogeneous mixture. Then, the powder mixture was pressed into a tablet for the IR spectrum. Data was collected in the wave number range of 400–4000 cm^−1^, with 24 accumulations, and then compared with the reported spectrum from the literature.

#### 3.3.2. UV Analysis

T4 was dissolved in 10 mL of water (0.5% ammonia), and was diluted with identical solution to 35 μg/mL. The sample solution was then analyzed by U3900, with the scan range of 190–400 nm.

#### 3.3.3. Mass Spectrum Analysis

T4 and structurally related organic impurities were analyzed with LC tandem Orbitrap Exploris^TM^ 240 and LC tandem Synapt G2 systems, respectively.

Orbitrap Exploris^TM^ 240 was operated in positive mode; the source voltage was set at +3500 V; the capillary temperature was maintained at 320 °C; the sheath gas and auxiliary gas were set to 25 arb. and 5 arb., respectively; the vaporizer temperature was set as 75 °C. For full-scan mode, the mass range of the orbitrap analyzer was set at *m*/*z* 400–1000, the resolution was set at 120,000, and the data type used was profile. For MS/MS analysis, the normalized collision energy for high-energy collision dissociation (HCD) was 30%; the isolation window was set at *m*/*z* 2; the resolution was set at 60,000; the data type used was profile. The mass accuracy of main compound was calculated based on the *m*/*z* values measured by Orbitrap Exploris^TM^ 240.

Synapt G2 was operated in resolution mode with a capillary voltage of 3000 V and a sample cone voltage of 25 V. MS full scan acquisitions were performed in the mass-to-charge (*m*/*z*) range of 400–1600, with a 1 s scan time. Afterward, transfer collision energy was set as 15 v to find their fragmented ions. LockSpray calibration was performed using a 2 mg/L LE solution in ACN/water (1/1). Data analysis was performed using MassLynx 4.1 software.

#### 3.3.4. ^1^H NMR Spectrum

T4 (~5 mg) was weighed into a glass vial and mixed with 0.6 mL dimethyl sulfoxide-*d*_6_ for complete dissolution. Afterward, the solution was pipetted to an NMR tube for characterization. ^1^H spectra were collected with a 30° flip angle, 32 s relaxation delay. Data was collected with 16 scans of 32 K data points. ^13^C spectra were collected with a 30° flip angle and 2 s relaxation delay. Data was collected with 1024 number of scans of 64 K data points. The head of NMR probe was maintained at 296 K. TopSpin 3.1 was used for data processing.

#### 3.3.5. Enantiomer Characterization

The enantiomer of L-T4 and D-T4 were analyzed with UPLC tandem Synapt G2. According to the previous studies [19,20,21], an Agilent Poroshell Chiral-CF (50 mm × 4.6 mm, 2.7 μm) was adopted as the analytical column. Meanwhile, specific optical rotation of T4 CRM was determined and calculated by Rudolph polarimeter. The sample-preparation process referred to the publication [22], and the details are described in the Supplementary Materials.

### 3.4. Test of Homogeneity and Stability

The homogeneity and stability tests were conducted using HPLC-UV analysis. All the packaged CRM units were assigned a unique numerical identifier, with the number corresponding to the order in which the units were to be dispensed. 

Fifteen units were randomly selected for between-bottle homogeneity testing, utilizing a random number table. From each bottle, three subsamples were prepared at a concentration of 1 mg/mL, which were then tested for homogeneity within the bottle. The data was analyzed using a single-factor analysis of variance (ANOVA).

The long-term stability of CRMs stored at −20 °C was assessed at intervals of 3, 12, 18, and 36 months. For short-term stability, units were kept at 4 °C and 20 °C for 0, 1, 2, 3, and 7 days. For each analysis, three units were randomly selected from the batches, and triplicate measurements were made at predetermined intervals.

During the investigations of homogeneity and stability, each sample underwent HPLC-UV analysis; the purity value was calculated by subtracting the moisture content measured at a predetermined time from the area normalization result, along with the non-volatile and volatile impurities determined during the purity assessment. Stability tests were evaluated using regression analysis.

### 3.5. MB Quantitative Experiments

MB method, the preferred method for purity determination, generally involves the methods for the accurate quantification of main component, moisture, volatile impurities, and residual inorganic impurities. The purity was calculated according to Equation (1) [29].

#### 3.5.1. Analysis of the Structurally Related Impurity 

T4 solution was injected and separated by a BEH C18 column (2.5 μm, 2.1 × 100 mm) with a flow rate of 0.25 mL/min. The mobile phase A was H_2_O (0.1% FA), and B was MeOH (0.1% FA). The gradient-elution process was as follows: 40% B for 1 min, rise to 60% B at 35 min, then rise to 85% B at 35.5 min, hold 85% B until 38 min, drop to 10% B at 38.5 min, rise to 40% B at 40 min, and hold 40% B until 45 min. Column temperature was 50 °C. The *m*/*z* of each impurity was acquired by a time-of-flight detector (Synapt G2, Manchester, UK). Meanwhile, the available impurities were purchased and analyzed using the identical LC-MS conditions to confirm the structure based on retention times and *m*/*z* of those ions.

The content of impurities was determined by the LC-UV method. When an impurity was identified, and its reference standards could be obtained, the content was calculated based on the external standard curve. If the reference standard was unavailable, a structural-related high-purity material was utilized as standard. However, when the structure and standard of an impurity cannot be obtained, the content of it was calculated based on the normalized peak area of all detected peaks. Because the peak area of impurities depended on the wavelength of UV detector, an uncertainty was introduced according to Standard JJF 1855-2020 [30].

#### 3.5.2. Analysis of the Water Impurity

Residual water in CRM was determined by Karl Fischer coulometric titration with a diaphragm-less electrode. Method conditions were polarization current, 5 μA; end voltage, 100 mV; and minimum titration time, 30 s. In detail, the water-content CRM was accurately weighed and introduced into the titration cell to check its accuracy. Then, T4 solid (about 15 mg) was precisely weighed and added for water determination. Six replicates were performed for T4 CRM.

#### 3.5.3. Analysis of the Inorganic Impurity

Both ICP-MS and IC analysis were adopted to determine inorganic impurity. In ICP-MS, the trace level of the common 76 elements was individually analyzed. By this method, T4 CRM solution was gravimetrically prepared at about 2 mg/g and infused for analysis. Parameters: Sampling depth, 10 mm; pump speed, 0.3 rps; RF power, 1550 W; dilution gas, 0.35 L/min; optional gas, 30% *v*/*v* O_2_ in Ar; nebulizer temperature, −5 °C; and helium carrier gas, 3.9 mL/min. A Dionex ICS-5000+ HPIC equipped with an anionic self-regenerating suppressor (ASRS 300) and a suppressed conductivity detector was used for CRM sample analysis. Chromatographic separation was accomplished with a Dionex IonPac AS19 column (250 mm × 4 mm; ThermoFischer) fitted with a guard column (50 mm × 4 mm; ThermoFisher). A linear gradient was used for separation of different ions: Initial 8 mM potassium hydroxide (KOH), 8–40 mM KOH from 0.01 to 40.00 min, 40–8 mM KOH from 40.00 to 40.01 min, and 8 mM KOH from 40.02 to 47.00 min. The IC flow rate was 1.0 mL/min, and the temperature of the column oven was set at 30 °C. The injection volume was 25 µL for each run.

#### 3.5.4. Analysis of the Residual Organic Solvent Impurity

About 15 mg of T4 material was weighed into a 20 mL headspace bottle, and 10 mL of water was added to analyze common 16 organic solvents. The equilibrium temperature, the equilibrium time, and the transmission line temperature were 90 °C, 30 min, and 110 °C, respectively. The chromatographic separation was performed on a DB-624 (60 m × 0.250 mm × 1.40 μm) with an argon gas flow rate of 1.5 mL/min. The initial temperature was set as 40 °C for 3 min, increased to 150 °C at a rate of 5 °C/min, and then increased to 240 °C at a rate of 40 °C/min. Full-scan monitoring mode was employed with an electron bombardment source, and the *m*/*z* range was from 29 to 300. The ion source and quadrupole temperatures were 230 °C and 150 °C.

### 3.6. ^1^H qNMR Quantitative Experiments

About 8 mg of T4 CRM and 4 mg of internal standard (ethylparaben) were accurately weighed. Then, the measurand and standard were co-dissolved in 0.6 mL DMSO-*d*_6_. The parameters of the instrument were the same as in Section 3.3.4. The mixture was transferred into an NMR tube for measurements. The purity of the T4 measurand was calculated according to the Equation (2) [29].

^1^H spectra were collected with a 30° flip angle, 32 s relaxation delay, and zg30 pulse sequence. Data was collected with 16 repetitions of 32 K complex points. The head of NMR probe was maintained at 298 K. TopSpin 3.1 was used for data processing.

### 3.7. Uncertainties

The homogeneity test ($u_{bb}$), short-term stability ($u_{sts}$), long-term stability ($u_{lts}$), and characterization ($u_{char}$) were found to be the main sources of uncertainty. To estimate $U_{char}$, the uncertainties from MB ($U_{MB}$) and qNMR ($U_{qNMR}$) were used.

## 4. Conclusions

This work outlines a comprehensive process for developing a T4-purity CRM, an essential biomarker for evaluating and diagnosing thyroid diseases. The qualitative characterization methods employed, including UV, IR, LC-MS/MS, ^13^C-NMR, and ^1^H-NMR, confirmed the structural identity of T4. Extensive research was conducted on all relevant impurities to ensure more accurate quantitative results. The methods of MB and qNMR determined T4 purity to be 94.90%, with an expanded uncertainty of 0.34%. Based on international standard documents, such as ISO 17511 [5] and ISO 15194 [31], since the well-characterized T4-purity CRM has been officially approved and designated as GBW09325 in China, it is feasible to utilize it as a primary standard for serum T4 CRM value assignment, thereby accelerating the progress of thyroid function standardization in China. Prior to this, applications involving the validation or control of the trueness of measured values in a given laboratory, as well as the evaluation of the performance of new measurement procedures, predominantly relied on the RMPs listed in the JCTLM database.

## Figures and Tables

**Table 2:** The results of value assignments by the two quantitative methods.

	$P_{o}$ (n = 6)	$X_{RS}$	$X_{W}$	$X_{V}$	$X_{NV}$	Purity	SD	*F* Value	$P_{char}$
MB	99.61%	1.006%	3.563%	0.0001%	0.132%	94.92%	0.095%	3.76	94.90%
qNMR	/	/	/	/	/	94.88%	0.049%		

**Table 3:** Uncertainty budget of T4 CRM (%).

$u_{MB}$	$u_{qNMR}$	$u_{char}$	$u_{bb}$	$u_{sts}$	$u_{lts}$	$u_{CRM}$	U
0.186	0.281	0.168	0.0047	0.01	0.005	0.168	0.336

## Data Availability

The data presented in this study are available on request from the corresponding author.

## Supplementary Materials

The following supporting information can be downloaded at https://www.mdpi.com/article/10.3390/molecules30132840/s1. Figure S1: IR spectrum of L-thyroxine (T4); Figure S2: UV absorption spectrum of T4; Figure S3: The main peak and impurities distribution when different elution times were employed; Figure S4: The main peak and impurities distribution when different column temperatures were employed; Figure S5: The impurities distribution when different UV wavelengths were employed (60 min/run, column temperature of 40 °C); Figure S6: The isotope peak distribution of T4_imp3 and T4_imp4; Figure S7: The fragmentation ions of T4_imp5; Figure S8: LC conditions optimization for separation of T4 enantiomer mixture. The extracted ion chromatography was acquired during isocratic elution under 40% hexane and 60% ethanol (A); 70% hexane and 30% ethanol (B); and 90% hexane and 10% ethanol, which were adopted respectively. Figure S9: Analysis of T4 enantiomer mixture based on the HPLC-DAD method during isocratic elution with 70% hexane and 30% ethanol. (A) The chromatogram of L-T4 CRM. (B) The ion chromatogram of D-T4 standard. (C) The chromatogram of the mixture of L-T4 CRM and D-T4 standard. Figure S10: Characterization results of the short-term stability of T4 CRM; Figure S11: Characterization results of the long-term stability of T4 CRM; Figure S12: The noise intensity of LC-UV baseline; Figure S13: The main component and structurally related impurities distribution with different injection amounts; Figure S14: UV absorption spectrum of each structural-related impurity; Figure S15: Standard curves plotted by the available standards of each impurity; Figure S16: The measurement results of water in T4 CRM (A, quality control; B, the trend of water content after unsealing the CRM unit); Figure S17: The measurement of counter ions in T4 CRM by ion chromatography approach; Figure S18: The measurement results of residual organic solvent by GC-MS (A, blank; B, mixture of standards and organic solvents; C, T4 CRM); Table S1: The measurement results of the specific optical rotation of different mixing ratios of L-T4 and D-T3; Table S2: Homogeneity test results of the T4 CRM by HPLC method; Table S3: Short-term stability test results of T4 CRM by HPLC; Table S4: Long-term stability test results of T4 CRM by HPLC; Table S5: The content of inorganic impurity measured by ICP-MS approach; Table S6: The content of inorganic impurity measured by ion chromatography approach (%).
