# Supplementary material for: Development of Certified Reference Material of L-Thyroxine by Using Mass Balance and Quantitative Nuclear Magnetic Resonance
Source: Molecules. 2025 Jul 2;30(13):2840. doi: 10.3390/molecules30132840 (PMC12251520; doi:10.3390/molecules30132840)
Supplement: Supplementary file 1 [file molecules-30-02840-s001.zip › molecules-3520069-supplementary.pdf]

Supplementary material

Development of certified reference material of L-thyroxine by using mass balance and quantitative nuclear magnetic resonance

Qiang Zhao[1,2,3], Weifei Zhang[2,3], Dan Song[2,3], Xirui Zhou[2,3], Xianjiang Li[2,3], Huan Yao[2,3], Hongmei Li[2,3], Wenjing Xing[1], Jian Ma[1*], Peng Xiao[2,3*]

1, Department of Immunology, Second Affiliated Hospital of Harbin Medical University, Harbin, China, Heilongjiang Province, 150081, China;

2, Division of Chemical Metrology and Analytical Science, National Institute of Metrology, Beijing, 100029, China;

3, Key Laboratory of Chemical Metrology and Applications on Nutrition and Health, State Administration for Market Regulation, Beijing 100029, China

*** Corresponding authors:**

Peng Xiao, Email: xiaop@nim.ac.cn

JianMa, Email: jma@hrbmu.edu.cn

Content

A. Experimental 2

B. Figures 3

C. Tables 14

A. Experimental

SI_3. Materials and Methods

SI_3.3.5 Enantiomer characterization

Three sets of solution were prepared by using 10 mL brown volumetric flasks. In specific, (1) a solution of L-T4 with a mass concentration of 3.3%, (2) a solution of L-T4 with a mass concentration of 3.3% and containing 0.01% (w/w) D-T4, and (3) a solution of L-T4 with a mass concentration of 3.3% and containing 0.1% (w/w) D-T4, for the analysis of the possible presence of D-T4 in the CRM.

The Rudolph polarimeter was preheated for 30 minutes, then to collect the optical rotation of levotar and dextrorotatory quality control samples under 546 nm. If the signal matches the quality control data, it indicates that the instrument is in a normal state. Thoroughly clean the tubing and then load the sample, with a volume of approximately 5 mL. Once the instrument stabilizes, provide three parallel measurement results and calculate the specific optical rotation $[\alpha^{20}]$ according the formula (S1):

$$[\alpha^{20}] = \frac{\alpha_{obs}}{lc} \qquad \text{(S1)}$$

Where $[\alpha^{20}]$ is the specific rotation at 20℃, α_{obs} is the measured value of optical rotation; l is the optical path length (mm), c is the sample concentration (g/100mL).

B. Figures

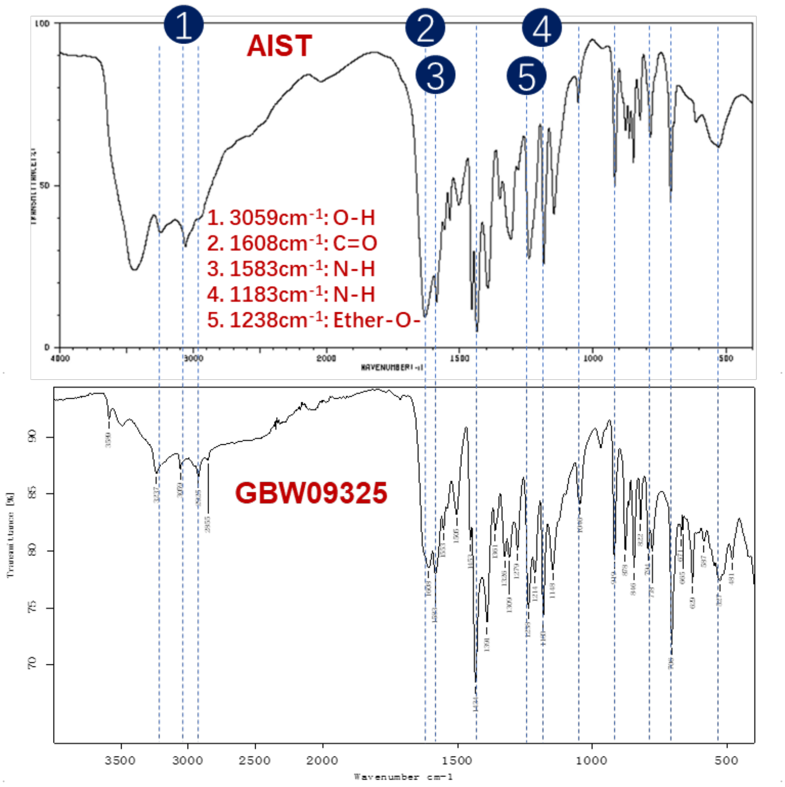

Figure S1
IR spectrum of L-thyroxine (T4)

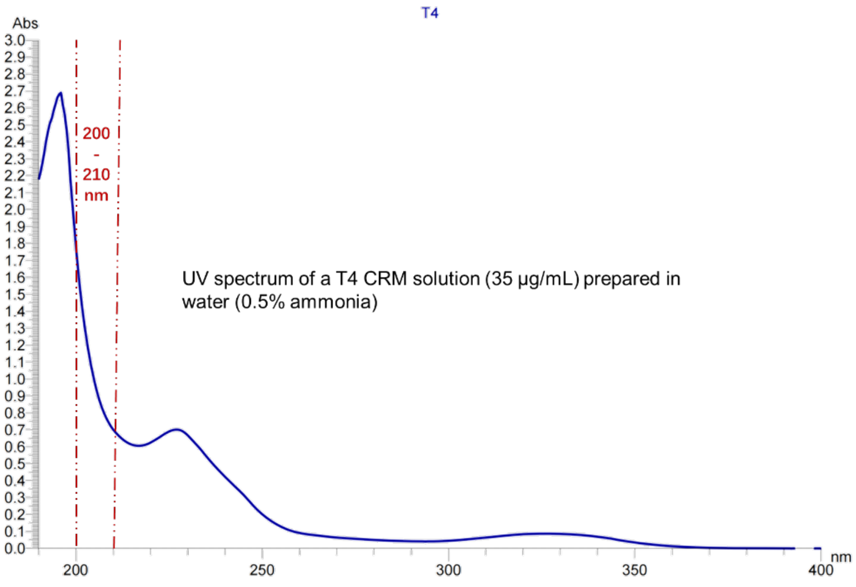

Figure S2 UV absorption spectrum of T4

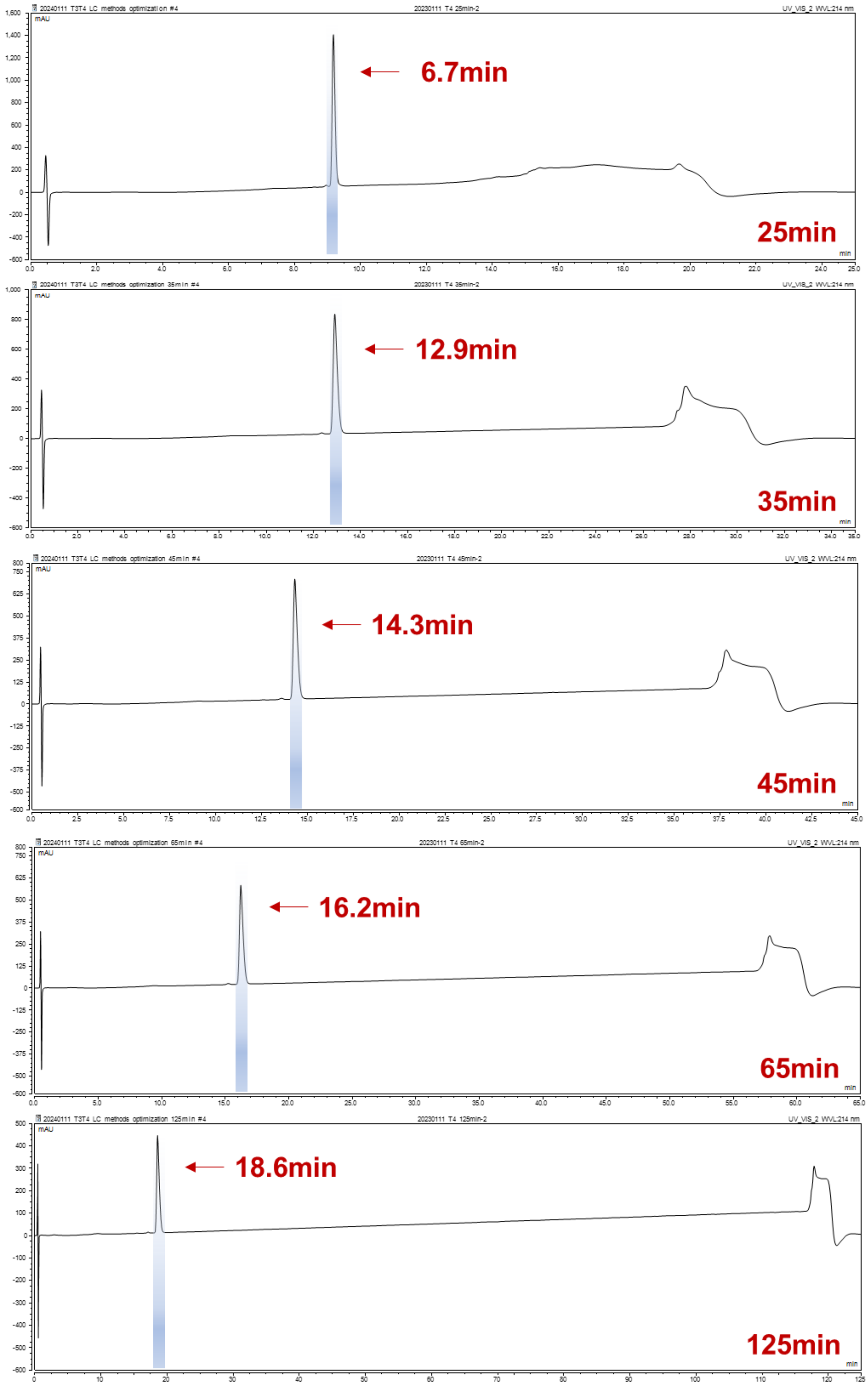

Figure S3

The main peak and impurities distribution when different elution times were employed

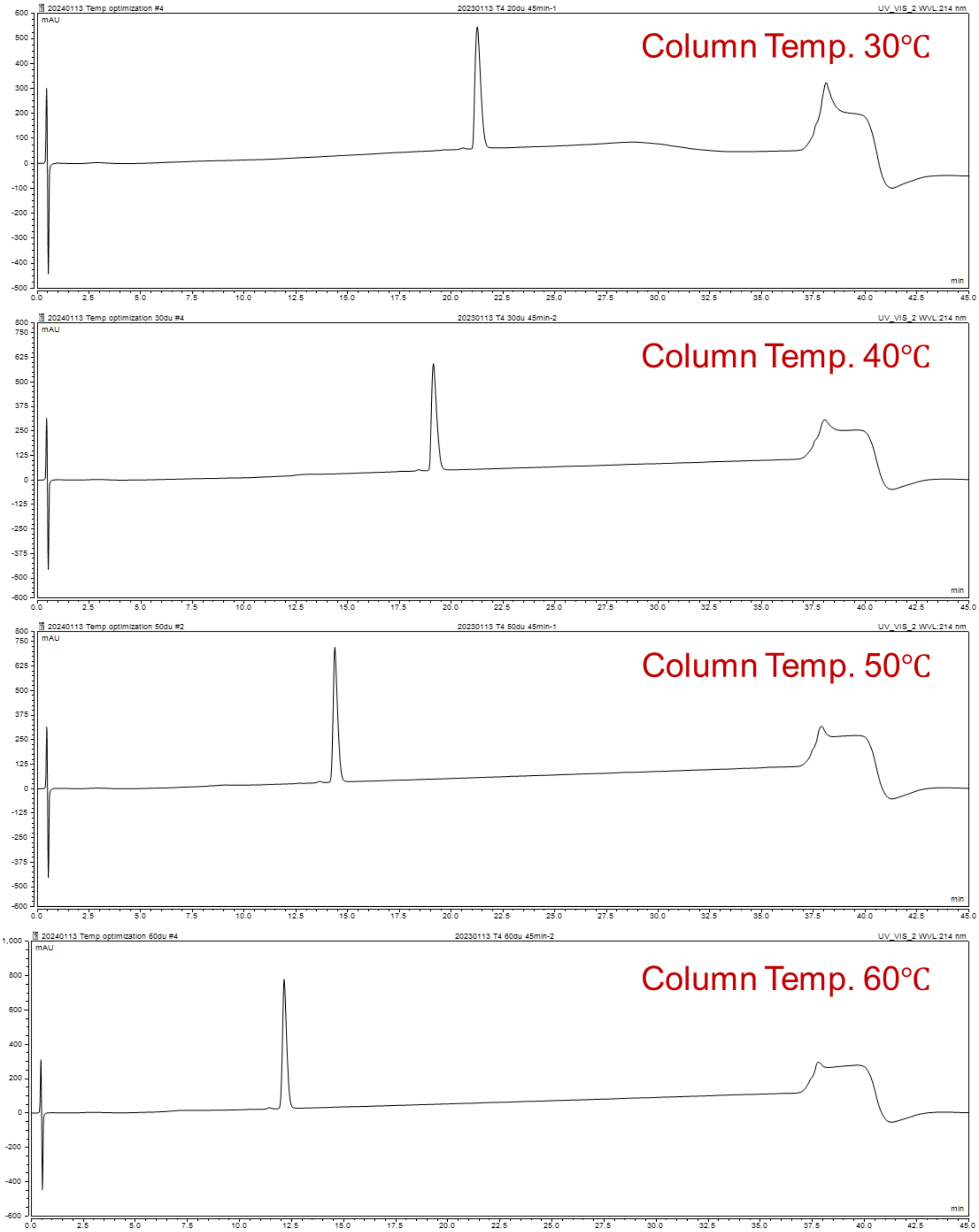

Figure S4
The main peak and impurities distribution when different column temperatures were employed

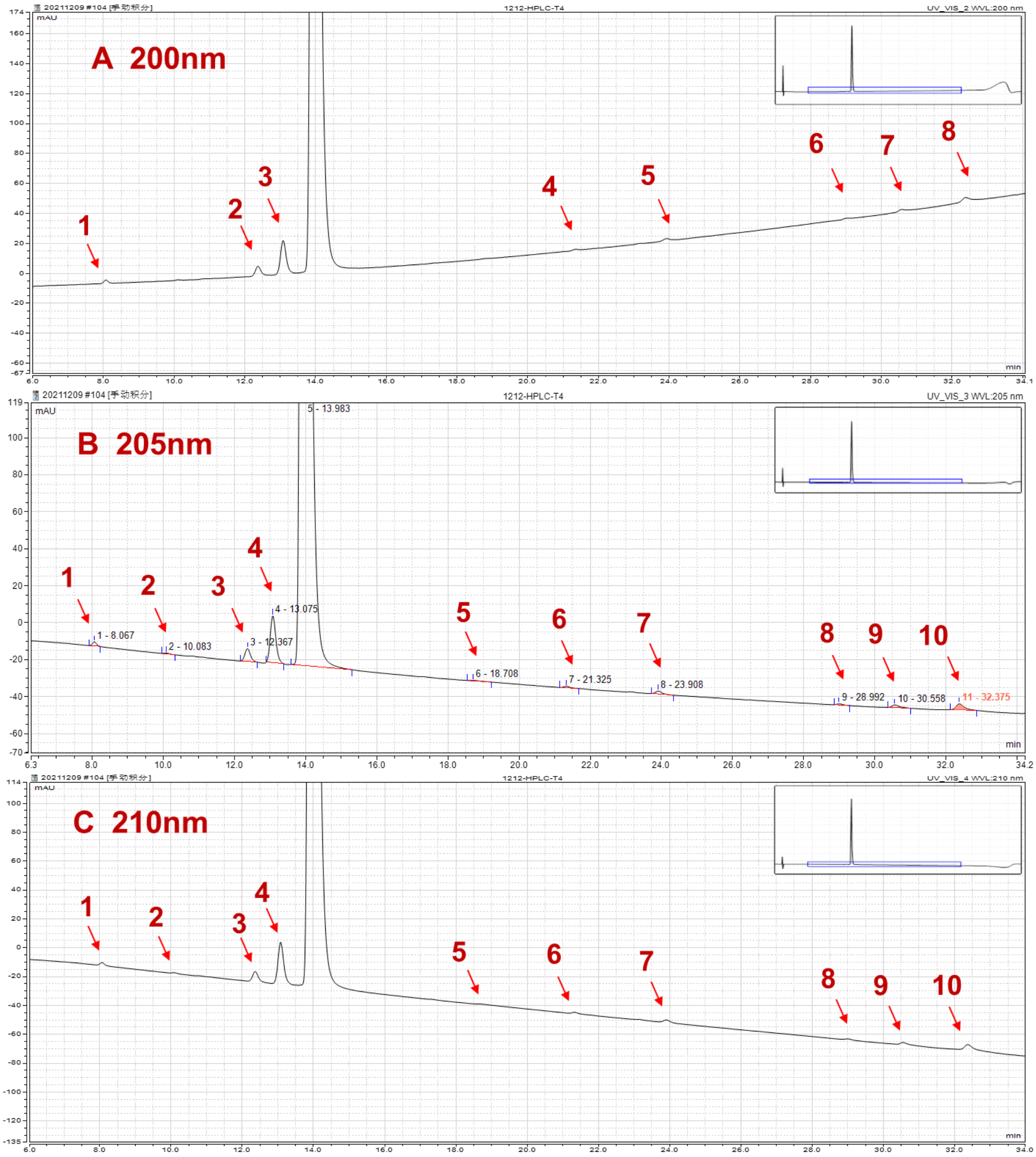

Figure S5

The impurities distribution when different UV wavelengths were employed (45 min per injection, column temperature of 50 °C)

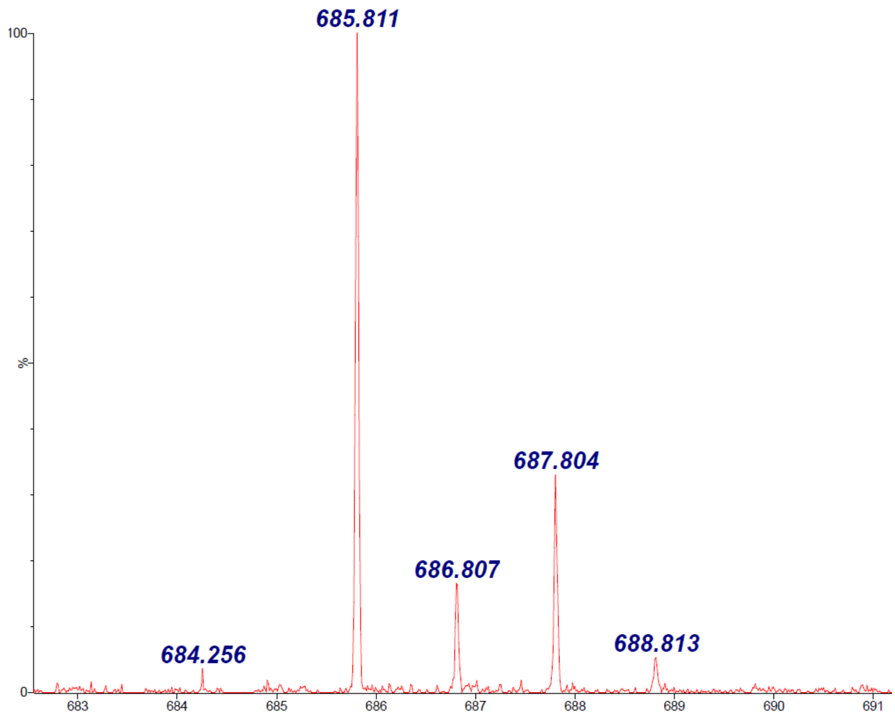

Figure S6
The isotope peak distribution of T4_imp3 and T4_imp4

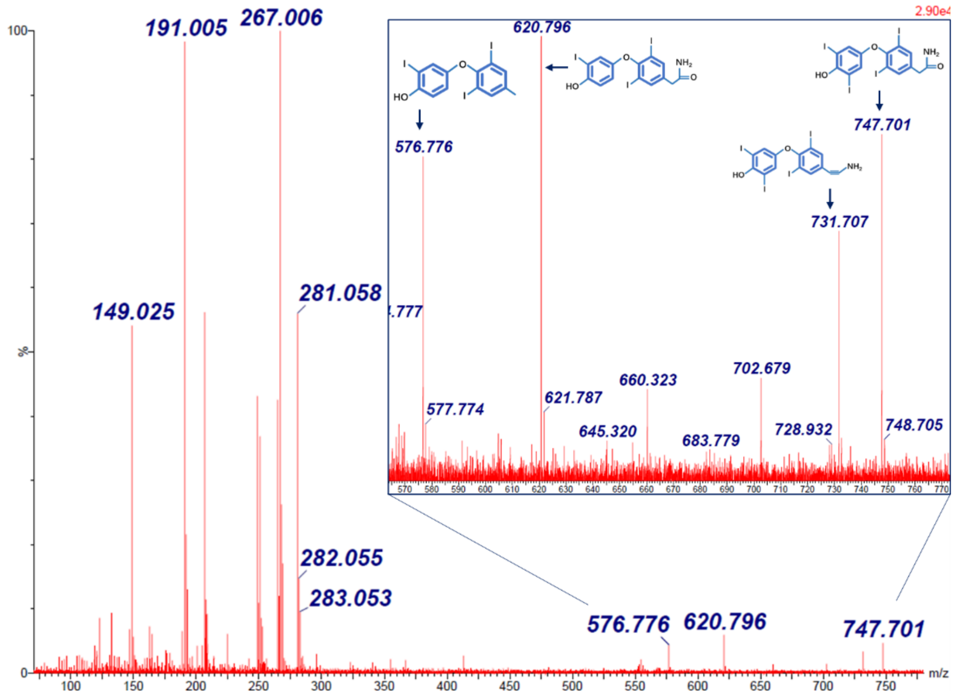

Figure S7
The fragmentation ions of T4_imp5

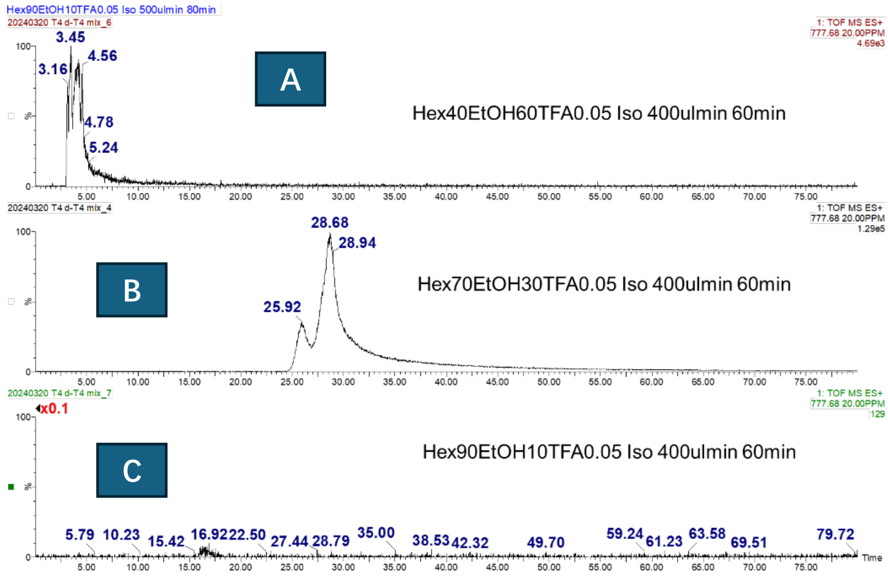

Figure S8

LC conditions optimization for separation of T4 enantiomer mixture. The extracted ion chromatography was acquired during isocratic elution under 40% hexane and 60% ethanol (A), 70% hexane and 30% ethanol (B), and 90% hexane and 10% ethanol, which were adopted respectively.

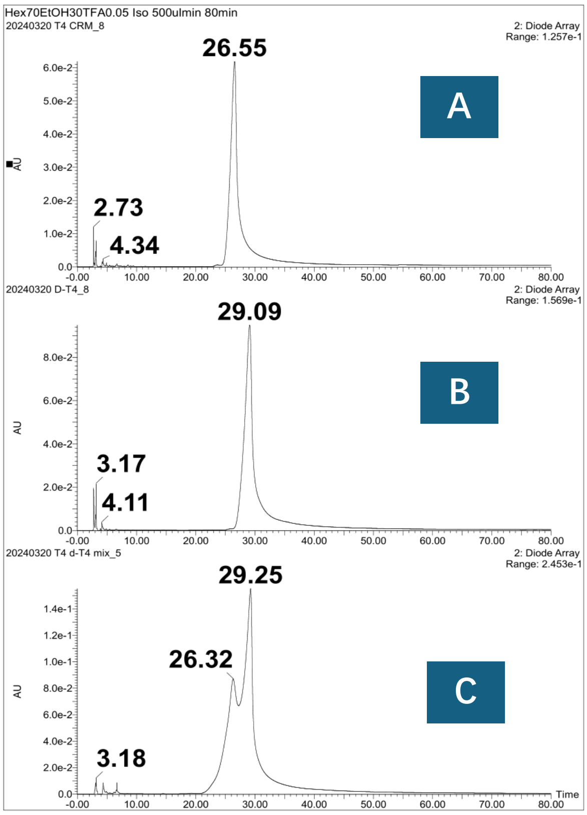

Figure S9

Analysis of T4 enantiomer mixture based on the HPLC-DAD method during isocratic elution with 70% hexane and 30% ethanol. (A) The chromatogram of L-T4 CRM; (B) The ion chromatogram of D-T4 standard; (C) The chromatogram of the mixture of L-T4 CRM and D-T4 standard.

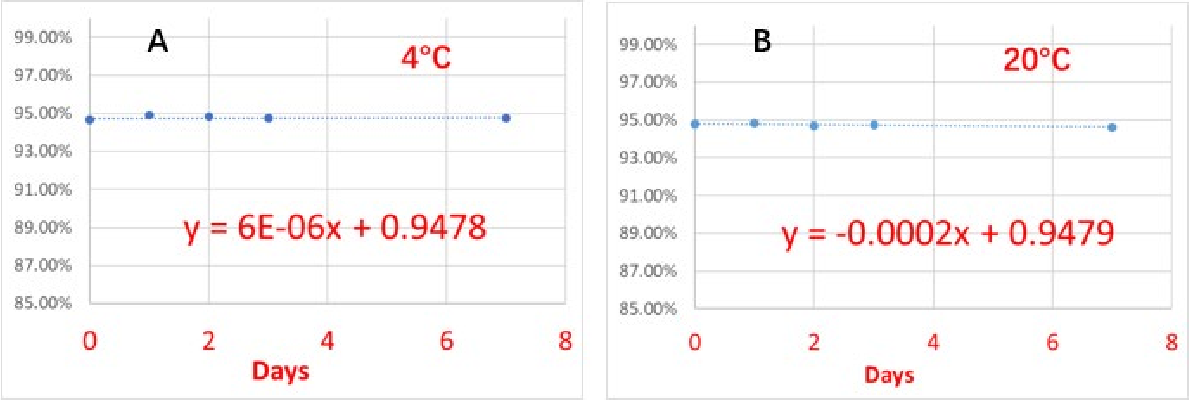

Figure S10
Characterization results of the short-term stability of T4 CRM

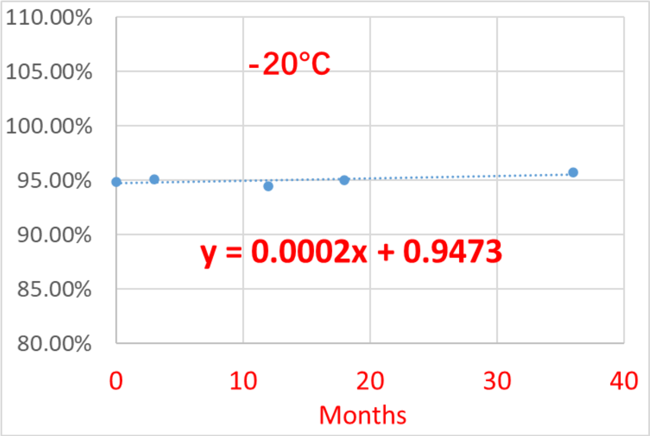

Figure S11
Characterization results of the long-term stability of T4 CRM

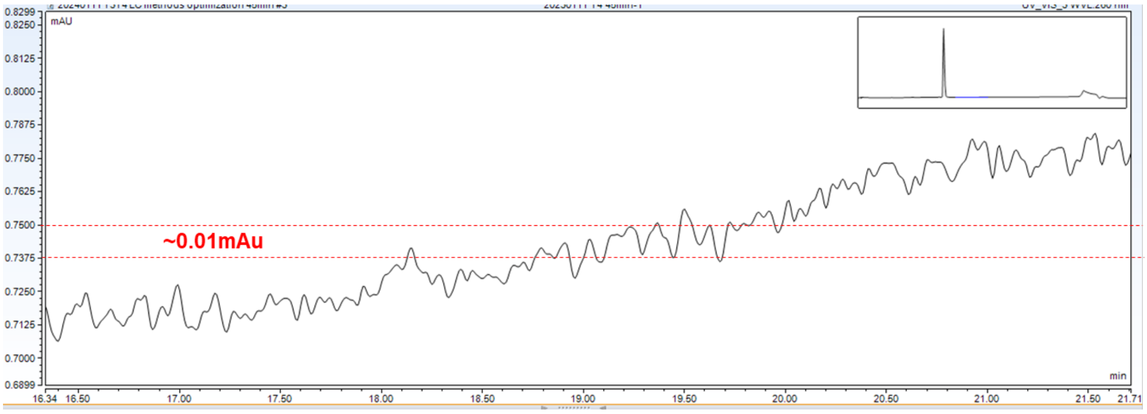

Figure S12
The noise intensity of LC-UV baseline

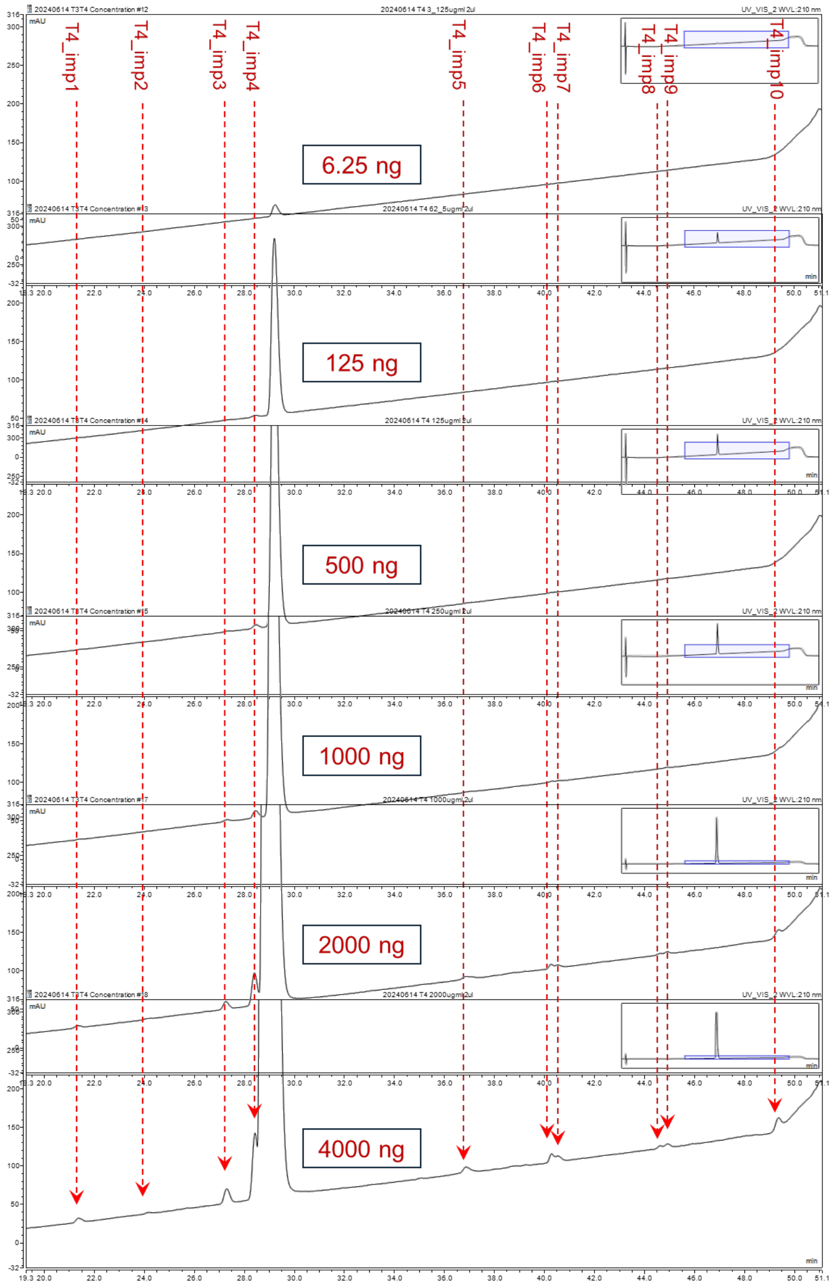

Figure S13

The main component and structurally related impurities distribution with different injection amounts (LC conditions only for this investigation as followed: sample was separated by a BEH C18 column (2.5 μm, 2.1×100 mm). The mobile phase A was H_2O (0.1% FA), and B was MeOH (0.1% FA). The gradient elution was as follows: 40% B for 5 min, rise to 70% B at 45 min, then rise to 85% B at 45.5 min, hold 85% B until 50 min, drop to 40% B at 50.5 min, and hold 40% B until 60 min. Column temperature was 22 °C ± 2 °C (room temperature))

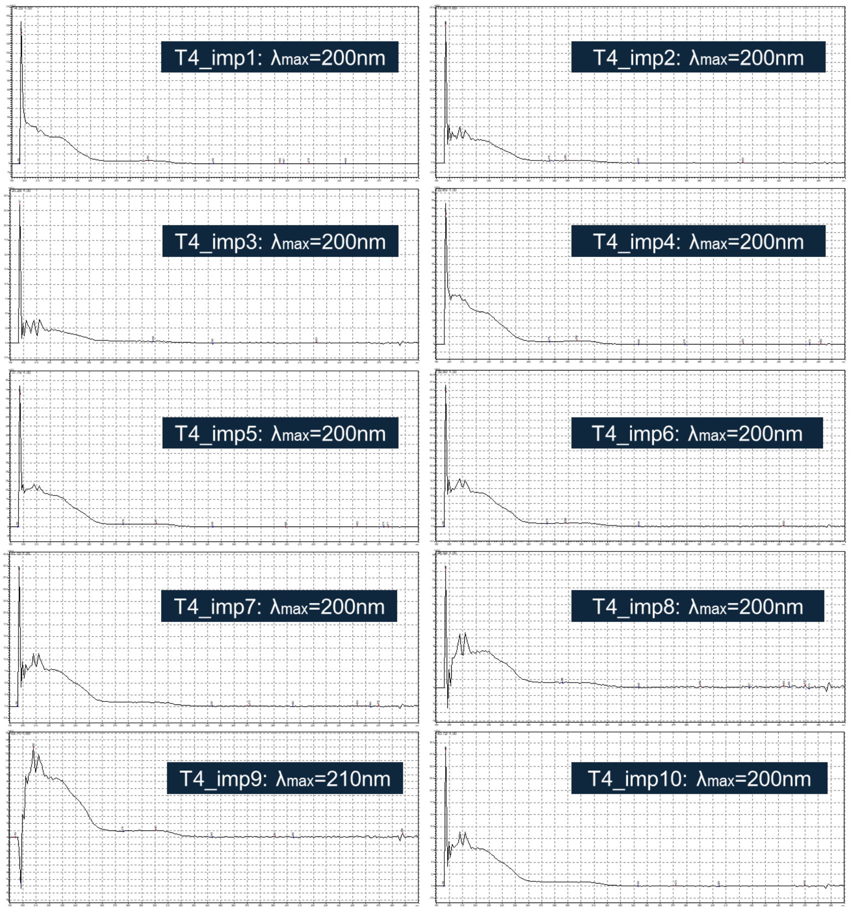

Figure S14

UV absorption spectrum of each structural-related impurity

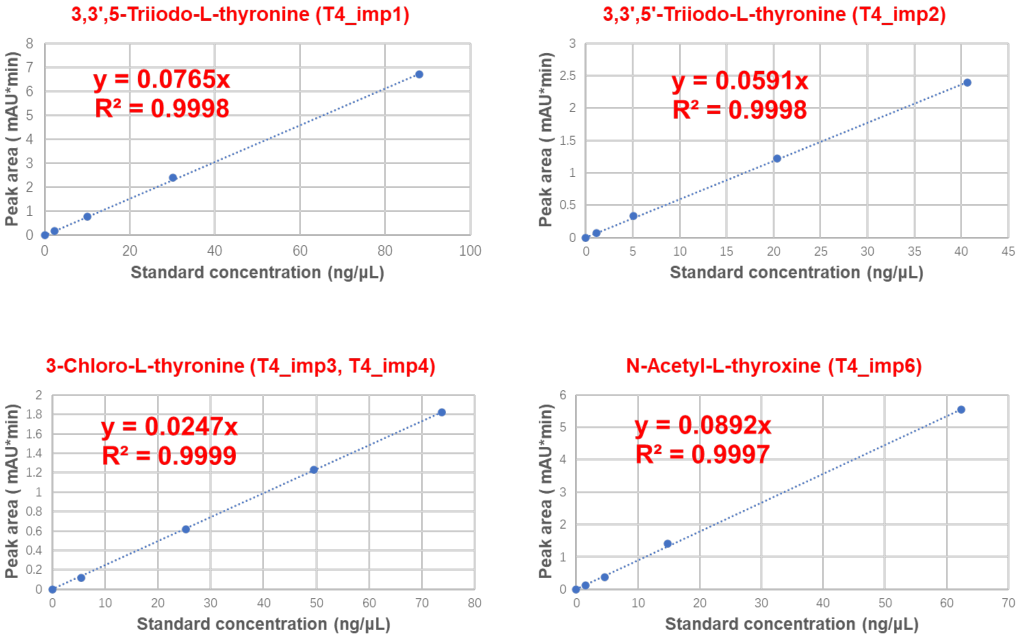

Figure S15

Standard curves plotted by the available standards of each impurity

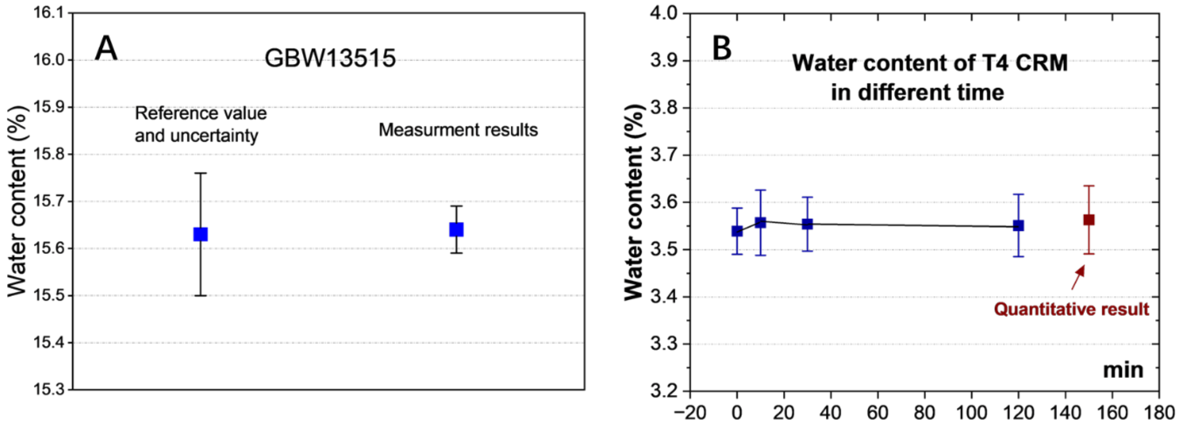

Figure S16
The measurement results of water in T4 CRM. (A, quality control; B, the trend of water content after unsealing the CRM unit)

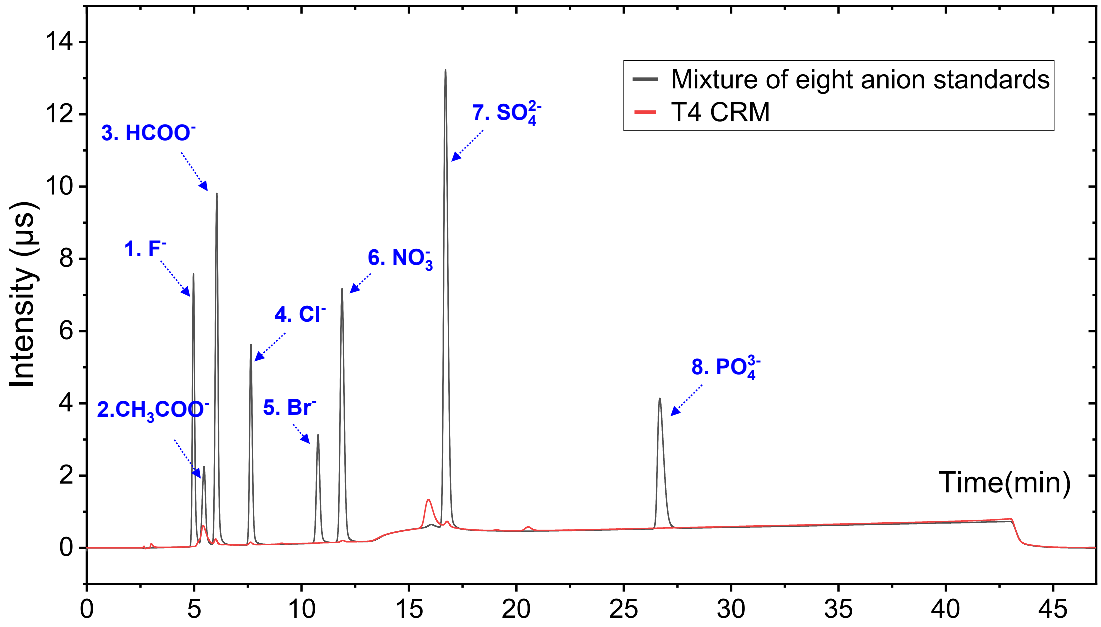

Figure S17
The measurement of counter ions in T4 CRM by ion chromatography approach

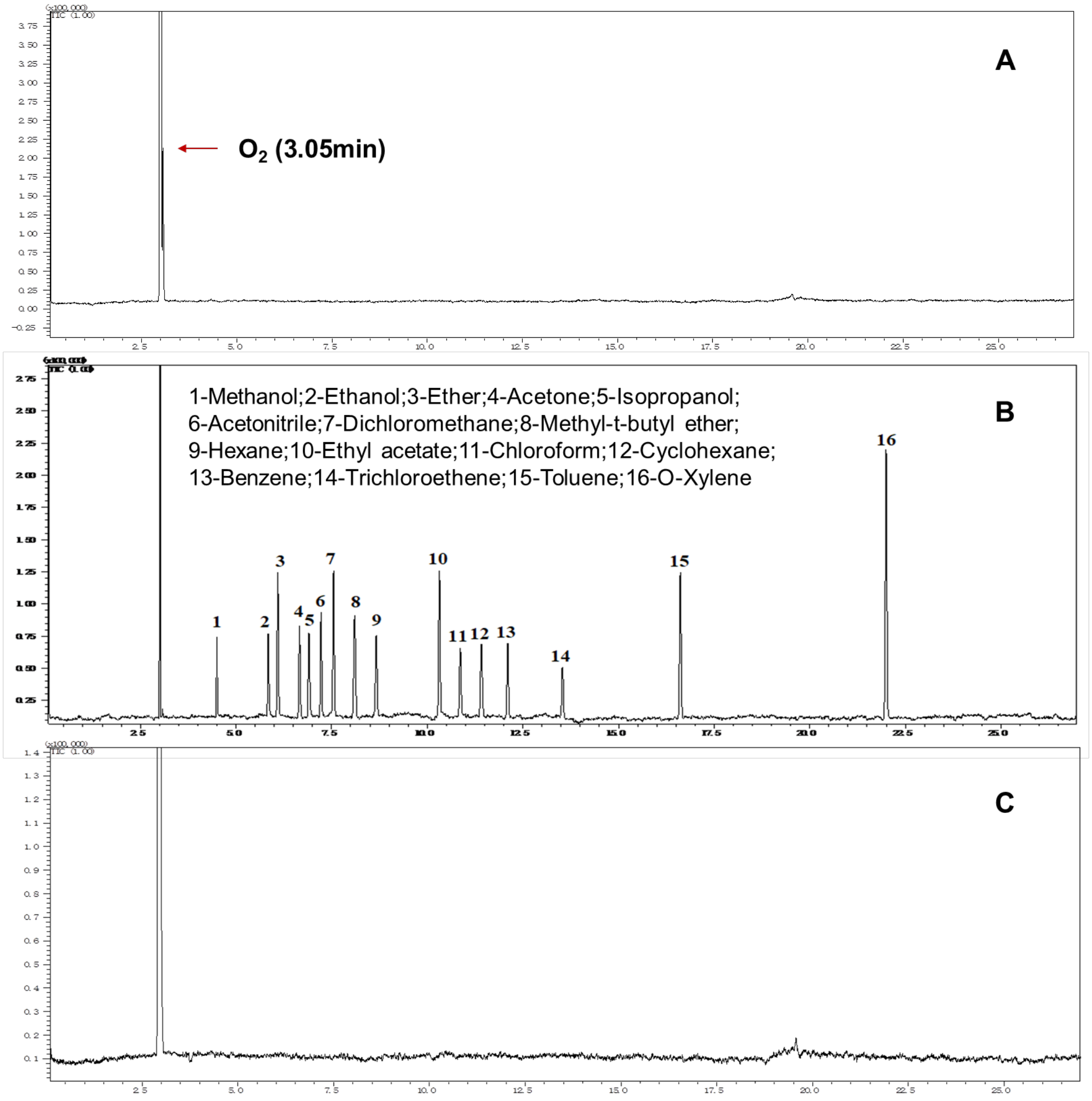

Figure S18
The measurement results of residual organic solvent by GC-MS (A, blank; B, mixture of standards and organic solvents; C, T4 CRM)

C. Tables

Table S1

The measurement results of the specific optical rotation of different mixing ratios of L-T4 and D-T3

	Sample 1			Sample 2			Sample 3		
	a solution of L-T4 with a mass concentration of 3.3%			a solution of L-T4 with a mass concentration of 3.3% and containing 0.01% (w/w) D-T4			a solution of L-T4 with a mass concentration of 3.3% and containing 0.1% (w/w) D-T4		
$[\alpha^{20}]$	-4.4	-4.4	-4.4	-4.4	-4.4	-4.4	-4.4	-4.4	-4.4

Table S2

Homogeneity test results of the T4 CRM by HPLC method

x_{ij}	Test results j=1	j=2	j=3	$\bar{x}_m$
i=1	94.84%	94.85%	94.88%	94.86%
i=2	94.80%	94.84%	94.85%	94.83%
i=3	94.93%	94.90%	94.88%	94.90%
i=4	94.89%	94.90%	94.95%	94.91%
i=5	94.91%	94.88%	94.82%	94.87%
i=6	94.83%	94.82%	94.83%	94.83%
i=7	94.90%	94.90%	94.95%	94.92%
i=8	94.83%	94.83%	94.89%	94.85%
i=9	94.84%	94.83%	94.84%	94.84%
i=10	94.91%	94.85%	94.85%	94.87%
i=11	94.85%	94.90%	94.92%	94.89%
i=12	94.89%	94.90%	94.88%	94.89%
i=13	94.79%	94.84%	94.75%	94.79%
i=14	94.90%	94.93%	94.96%	94.93%
i=15	94.94%	94.95%	94.91%	94.93%
	$\bar{\bar{x}} = \frac{1}{N}\sum_{i=1}^{m}\sum_{j=1}^{n} x_{ij}$=94.87%			N=45
	Sum of differences between-bottle, Q_1=2.46522E-06		Sum of differences within-bottle, Q_2=2.59333E-06	
	Degree of freedom between-bottle, v_1=14		Degree of freedom within-bottle, v_2=30	
	Between-bottle variance, $S_1^2=Q_1/v_1$= 1.76087E-07		Within-bottle variance, $S_2^2=Q_2/v_2$= 8.64444E-08	

$F= S_1^2/S_2^2=2.03$	$F_{0.05}(14, 30)$ =2.31 (95% confidence level)

Table S3
Short-term stability test results of T4 CRM by HPLC

Day	4 ℃	20 ℃
0	94.66%	94.76%
1	94.90%	94.83%
2	94.85%	94.71%
3	94.74%	94.75%
7	94.77%	94.63%
Average	94.78%	94.74%
β_1	-3.80952E-06	-4.34921E-05
β_0	0.947898571	0.948132857
s^2	0.000195	0.000205
$s(\beta_1)$	0.001244504	0.001276569
$t_{0.95,3}$	3.18	
$t_{(0.95,3)} \cdot s(\beta_1)$	0.003957524	0.00405948
Results	$\|\beta_1\| < t_{0.95,3} \cdot s(\beta_1)$	$\|\beta_1\| < t_{0.95,3} \cdot s(\beta_1)$

Table S4
Long-term stability test results of T4 CRM by HPLC

Random bottle		0 month	3 months	12 months	18 months	36 months
1#	Subsample_1	94.71%	94.90%	94.05%	94.83%	95.65%
	Subsample_2	94.95%	95.02%	94.85%	94.91%	95.88%
2#	Subsample_1	95.12%	95.19%	94.74%	95.04%	96.11%
	Subsample_2	94.94%	95.07%	94.52%	95.01%	95.38%
3#	Subsample_1	94.57%	95.03%	95.02%	94.75%	95.50%
	Subsample_2	94.76%	94.36%	93.52%	94.87%	95.70%
Average		94.84%	95.03%	94.75%	94.90%	95.02%
Standard deviation		0.198%	0.096%	0.190%	0.110%	0.282%
Results		β_1=0.0003, β_0=0.947, s=0.008471, s(β_1)=0.00028, $t_{0.95,3}$=3.18, $t \cdot s(\beta_1)$=0.000876247				
Conclusion		$\|\beta_1\| < t \cdot s(\beta_1)$, the slope is not significant, and the sample's value remains stable after being stored at -20 ℃ for 36 months				

Table S5

The content of inorganic impurity measured by ICP-MS approach

Elements	Content (μg/L)	Elements	Content (μg/L)
^{7}Li	/	^{115}In	/
^{9}Be	/	^{118}Sn	/
^{11}B	/	^{121}Sb	/
^{24}Mg	/	^{125}Te	/
^{27}Al	/	^{127}I	/
^{29}Si	/	^{132}Xe	/
^{31}P	/	^{133}Cs	/
^{35}Cl	/	^{137}Ba	/
^{39}K	419.5	^{139}La	/
^{44}Ca	199.9	^{140}Ce	/
^{45}Sc	/	^{141}Pr	/
^{47}Ti	/	^{146}Nd	/
^{51}V	/	^{147}Sm	/
^{52}Cr	/	^{153}Eu	/
^{55}Mn	/	^{157}Gd	/
^{56}Fe	/	^{159}Tb	/
^{59}Co	/	^{163}Dy	/
^{60}Ni	/	^{165}Ho	/
^{65}Cu	0.162	^{166}Er	/
^{66}Zn	/	^{169}Tm	/
^{69}Ga	/	^{172}Yb	/
^{72}Ge	/	^{175}Lu	/
^{75}As	/	^{178}Hf	/
^{79}Br	0.896	^{181}Ta	/
^{82}Se	/	^{182}W	/
^{84}Kr	/	^{185}Re	/
^{85}Rb	/	^{189}Os	/
^{88}Sr	/	^{193}Ir	/
^{89}Y	/	^{195}Pt	/
^{90}Zr	/	^{197}Au	/
^{93}Nb	/	^{202}Hg	0.0503
^{95}Mo	/	^{205}Tl	/
^{99}Tc	/	^{208}Pb	0.011

^{101}Ru	/	^{209}Bi	/
^{103}Rh	0.006	^{232}Th	/
^{105}Pd	0.9003	^{237}Np	/
^{107}Ag	/	^{238}U	/
^{111}Cd	/	^{239}Pu	/
**Total**	0.00062142 mg/g (0.000062142%)		

Table S6

The content of inorganic impurity measured by ion chromatography approach (%)

Counter ions	Acetate	Formate	Chloridion	Nitrate	Sulfate
Contents (ppm)(n=3)	1.041	0.065	0.041	0.035	0.137
Total			0.132%		
